# Long-Term Survival and Life-Sustaining Device Use in Survivors of First and Second Out-of-Hospital Cardiac Arrest: Retrospective Cohort Study

**DOI:** 10.2196/90416

**Published:** 2026-06-02

**Authors:** Chih-Hsien Lin, Cheng-Yi Fan, Edward Pei-Chuan Huang, Chih-Wei Sung

**Affiliations:** 1 Department of Emergency Medicine National Taiwan University Hospital Hsin-Chu Branch Hsinchu Taiwan; 2 Department of Emergency Medicine College of Medicine National Taiwan University Taipei Taiwan; 3 Institute of Molecular Medicine, National Tsing Hua University Hsinchu Taiwan

**Keywords:** out-of-hospital cardiac arrest, second cardiac arrest, life-sustaining device, risk stratification, National Health Insurance Research Database

## Abstract

**Background:**

Long-term surveillance of out-of-hospital cardiac arrest (OHCA) survivors is increasingly important, but patients who survive a second OHCA are rarely characterized because of the scarcity of such cases. The nationwide claims-based health data provide an opportunity to identify this uncommon survivor population and evaluate postdischarge outcomes at the population level. Understanding the prognosis and care needs of second-time OHCA survivors may help inform postarrest surveillance, risk stratification, and long-term care planning.

**Objective:**

This study aimed to compare the clinical characteristics of adult patients who survived a single OHCA versus those who survived a second OHCA during a 5-year follow-up and assess and contrast their 5-year survival outcomes.

**Methods:**

We conducted a nationwide retrospective cohort study using data from the National Health Insurance Research Database (2010-2020) in Taiwan. Survivors at discharge were classified as 1-OHCA survivors, defined as patients who survived exactly 1 OHCA episode and had no documented recurrent OHCA during follow-up, or 2-OHCA survivors, defined as patients who experienced exactly 2 documented OHCA episodes and survived to discharge after both events. Patients with more than 2 OHCA episodes were excluded. Postdischarge survival was measured from the qualifying discharge date: discharge after the first OHCA for 1-OHCA survivors and discharge after the second OHCA for 2-OHCA survivors. Variables included demographics, comorbidities, health care use, and life-sustaining device status. Five-year mortality was analyzed using the Kaplan-Meier and log-rank tests. Adjusted hazard ratios were derived from multivariable Cox models. Device use patterns were compared using Cochran-Armitage trend tests.

**Results:**

Among 239,929 OHCA cases, 229,047 were eligible; 15,617 survived to hospital discharge. Five-year mortality was substantially higher among 2-OHCA survivors than among 1-OHCA survivors (191/201, 95% vs 6850/14,494, 47.3%; log-rank *P*<.001). In patients with 1 OHCA, mortality increased with age, male sex, and nasogastric tube use, whereas >3 outpatient visits, Foley catheter use, and tracheostomy or ventilation were associated with lower observed postdischarge mortality. In the 2-OHCA group, the rate of device-free status was lower, while the triple-device use rate was higher.

**Conclusions:**

In this nationwide claims-based surveillance study, patients who survived discharge after a second OHCA represented a rare and clinically vulnerable postarrest population with poor subsequent 5-year survival and greater life-sustaining device dependence. Device-related variables should be interpreted as claims-based markers of postdischarge dependency, care setting, survivorship, and care intensity rather than evidence that device placement itself improves survival. As 2-OHCA survivor status is conditional on survival to a second OHCA and discharge after that event, these findings should be interpreted as descriptive and hypothesis-generating rather than causal. Population-level health data may support long-term postarrest surveillance and care planning for rare OHCA survivor populations.

## Introduction

Out-of-hospital cardiac arrest (OHCA) remains a global health burden with persistently low survival rates, despite advancements in emergency care. For example, in the United States, survival to hospital discharge following emergency medical service–treated OHCA is approximately 9% to 10% [1,2], while in Asia, the survival rate ranges between 3% and 8% [3,4]. With the increasing implementation of guideline-based resuscitation, a rising number of OHCA patients survive until hospital discharge. These patients require long-term follow-up [5].

Recognizing this clinical shift, the 2020 American Heart Association Guidelines introduced “recovery” as the sixth link in the chain of survival, emphasizing the importance of long-term surveillance and care for OHCA survivors [6]. However, there has been limited attention to the long-term trajectories of these survivors, particularly those who experience second OHCA events postdischarge [7,8]. Of the OHCA survivors, 6% to 10% experience a second cardiac arrest, often within the first year of discharge [7,9]. While a second OHCA is associated with exceedingly high mortality, data regarding patients who suffer a second OHCA and survive remain scarce. No study has systematically compared the survival patterns and baseline characteristics of patients who survive 1 OHCA with those who survive 2.

Survivors of 1-OHCA face increased risks of neurological injury, cardiovascular events, and long-term mortality [10,11]. Surviving 2 OHCA episodes may lead to greater physiological compromise, including cumulative hypoxic brain injury and myocardial dysfunction, which could profoundly influence long-term survival. Understanding whether the trajectories of these 2 groups diverge in terms of both survival curves and comorbidity profiles is crucial for planning postresuscitation care, developing rehabilitation strategies, and allocating resources.

Identifying patients who survive 2 OHCA events poses a substantial methodological challenge owing to the rarity of such events. Indeed, most single-center or regional databases lack sufficient sample sizes to permit meaningful analyses. To overcome this limitation, we leveraged Taiwan’s National Health Insurance Research Database (NHIRD), a comprehensive nationwide claims-based database that captures nearly all medical events among 23 million residents [12].

From a public health surveillance perspective, 2-OHCA survivors represent a rare but high-risk population that is difficult to characterize using single-center registries or conventional clinical cohorts. Nationwide administrative claims databases can function as longitudinal health surveillance infrastructure by enabling identification of uncommon survivor populations, monitoring of long-term mortality, and assessment of postdischarge care dependency at the population level. Using a nationwide claims-based surveillance database, this study aimed to compare clinical characteristics, health care use, life-sustaining device dependence, and subsequent postdischarge survival between adult 1-OHCA and 2-OHCA survivors.

## Methods

### Study Design and Setting

This retrospective study used data from the Taiwan NHIRD. Established in 1995 under the nation’s single-payer National Health Insurance program, the NHIRD contains comprehensive claims information for more than 23 million citizens, covering >99% of Taiwan’s population [13]. These data include registries of insured individuals, diagnostic codes, outpatient and inpatient visits, prescriptions, and other relevant health care variables, forming a powerful resource for population-based and epidemiological research [13,14]. Additionally, the NHIRD can be linked to disease-specific registries and other government datasets, substantially augmenting real-world evidence generation [15]. To protect patient privacy, rigorous deidentification procedures and ethical oversight protocols are in place, ensuring that individual identities remain anonymous while allowing researchers to conduct robust retrospective analyses.

### Ethical Considerations

This study was approved by the institutional review board of the National Taiwan University Hospital (approval number 202502001RINC). The requirement for informed consent was waived by the institutional review board because this was a retrospective analysis using anonymized data. To protect patient privacy and confidentiality, all data used in the analysis were anonymized before access by the study investigators, and no directly identifiable personal information, such as names, medical record numbers, or other individual identifiers, was included in the analytic dataset or reported in the manuscript. The data were used solely for the purposes approved by the institutional review board. Because this study involved secondary analysis of existing anonymized data and did not involve direct contact with patients or prospective participant enrollment, no compensation was provided to participants. All study procedures adhered to relevant ethical standards and regulations, including the principles outlined in the Declaration of Helsinki [16].

### Study Population and Eligibility Criteria

We identified patients who experienced OHCA between 2010 and 2020 by querying the NHIRD. To be included, individuals had to present to the emergency department (ED) with a triage level of 1 and documented *International Classification of Diseases* (*ICD*) codes reflecting cardiac arrest (*ICD-9*: 427.4, 427.5, 798, 798.1, 798.2, 798.9, and 799.9; *ICD-10*: I46, I46.2, I46.8, I46.9, and I49.0). Both *ICD-9* and *ICD-10* were used owing to the nationwide transition from the former coding system to the latter, which occurred after 2016. We excluded cases in which OHCA was attributed to traumatic events, individuals aged <18 years, and instances in which the recorded date of death preceded the OHCA. These criteria were designed to focus on the analysis of nontraumatic OHCA and ensure proper assessment of the risk factors for subsequent OHCA among survivors.

### Cohort Definition: 1-OHCA and 2-OHCA Survivors

First-event OHCA survivors (1-OHCA survivors) were defined as patients who had exactly 1 documented nontraumatic OHCA episode during the study period, survived to hospital discharge after that episode, and had no subsequent documented OHCA during follow-up. Second event OHCA survivors (2-OHCA survivors) were defined as patients who had exactly 2 distinct documented nontraumatic OHCA episodes during the study period and survived to hospital discharge after both events. In this study, survival to hospital discharge was operationalized as the absence of a death record in the NHIRD at the time of discharge. These definitions ensured that the 2 analytic groups were mutually exclusive: patients classified as 2-OHCA survivors were not counted as 1-OHCA survivors. Patients with 3 or more documented OHCA episodes were not included in the analytic cohort because such cases were exceedingly rare, typically limited to single-digit counts, and were subject to NHIRD privacy protection restrictions for rare and potentially identifiable cases. Accordingly, we used the term 2-OHCA survivors, rather than ≥2-OHCA survivors, throughout the revised manuscript to accurately reflect the analytic cohort.

### Covariates and Operational Definitions

For all covariates used in the mortality models, the anchor time was defined as the discharge date after the qualifying OHCA event: discharge after the first OHCA for 1-OHCA survivors and discharge after the second OHCA for 2-OHCA survivors. All covariates were entered into the Cox models as fixed baseline variables and were not updated during follow-up.

The variables were grouped into 4 main categories—demographic characteristics, comorbidities, health care use, and daily functional status—to characterize differences between 1-OHCA and 2-OHCA survivors and to evaluate factors associated with postdischarge mortality within each survivor group. Demographic characteristics included age and sex. Comorbidities included diabetes mellitus, hypertension, hepatitis, chronic kidney disease, asthma, peptic ulcer disease, heart disease, cancer, cerebrovascular disease, hyperlipidemia, chronic obstructive pulmonary disease, and liver cirrhosis, each identified using insurance claims and prescription histories before the anchor date. Health care use variables were assessed using prespecified look-back windows before the anchor date, including intensive care unit (ICU) admission within the previous 365 days, renal dialysis for more than 3 consecutive months, more than 3 outpatient visits within the previous 30 days, and more than 2 ED or inpatient visits within the previous 30 days. Daily functional status was approximated using claims-based indicators of nasogastric tube placement, Foley catheter use, and tracheostomy with long-term mechanical ventilation before or at the qualifying discharge, which served as surrogate markers of functional dependency and ongoing care needs at the start of mortality follow-up.

### Statistical Analysis

Descriptive statistics were used to summarize baseline characteristics, health care use, comorbidities, and life-sustaining device use. Continuous variables were compared using Student’s *t* test, and categorical variables were compared using the chi-square test or Fisher exact test, as appropriate. We performed Kaplan-Meier analyses to describe postdischarge survival trajectories among 1-OHCA and 2-OHCA survivors, and differences in survival distributions were assessed using the log-rank test [17]. For 1-OHCA survivors, follow-up began at discharge after the first OHCA; for 2-OHCA survivors, follow-up began at discharge after the second OHCA. As the 2-OHCA survivor status is conditional on surviving the recurrent event and discharge, the survival curves were interpreted as descriptive postdischarge prognostic comparisons rather than causal estimates of the effect of recurrent OHCA.

To identify factors associated with postdischarge mortality within each survivor group, we first performed univariate Cox proportional hazards regression analyses for candidate variables. Variables with a univariate *P* value <.10 were considered for inclusion in the multivariable Cox proportional hazards models. Clinically important covariates, including age and sex, were also considered for inclusion when appropriate. The 1-OHCA and 2-OHCA groups were modeled separately, and adjusted hazard ratios (aHRs) with 95% CIs were reported. As the 2-OHCA survivor subgroup was relatively small, we used a parsimonious modeling strategy to reduce overfitting and model instability. Variables with sparse distributions, complete or near-complete separation, or unstable estimates were not forced into the final 2-OHCA model. The proportional hazards assumption was assessed for retained covariates using Schoenfeld residuals and graphical inspection of log-minus-log survival plots. No major violation of the proportional hazard assumption was identified [18].

On the basis of clinical considerations, ICU admission and more than 2 ED or ward hospitalizations were not included in the multivariable models because these variables were closely related to disease severity, ventilator use, and subsequent care intensity, and therefore could introduce collinearity or overadjustment. Device use patterns were compared between groups using Cochran-Armitage trend tests. All statistical analyses were performed using SAS software (version 9.4; SAS Institute). Statistical significance was defined as a 2-sided *P* value of <.05.

## Results

### Overview

**Figure 1:**
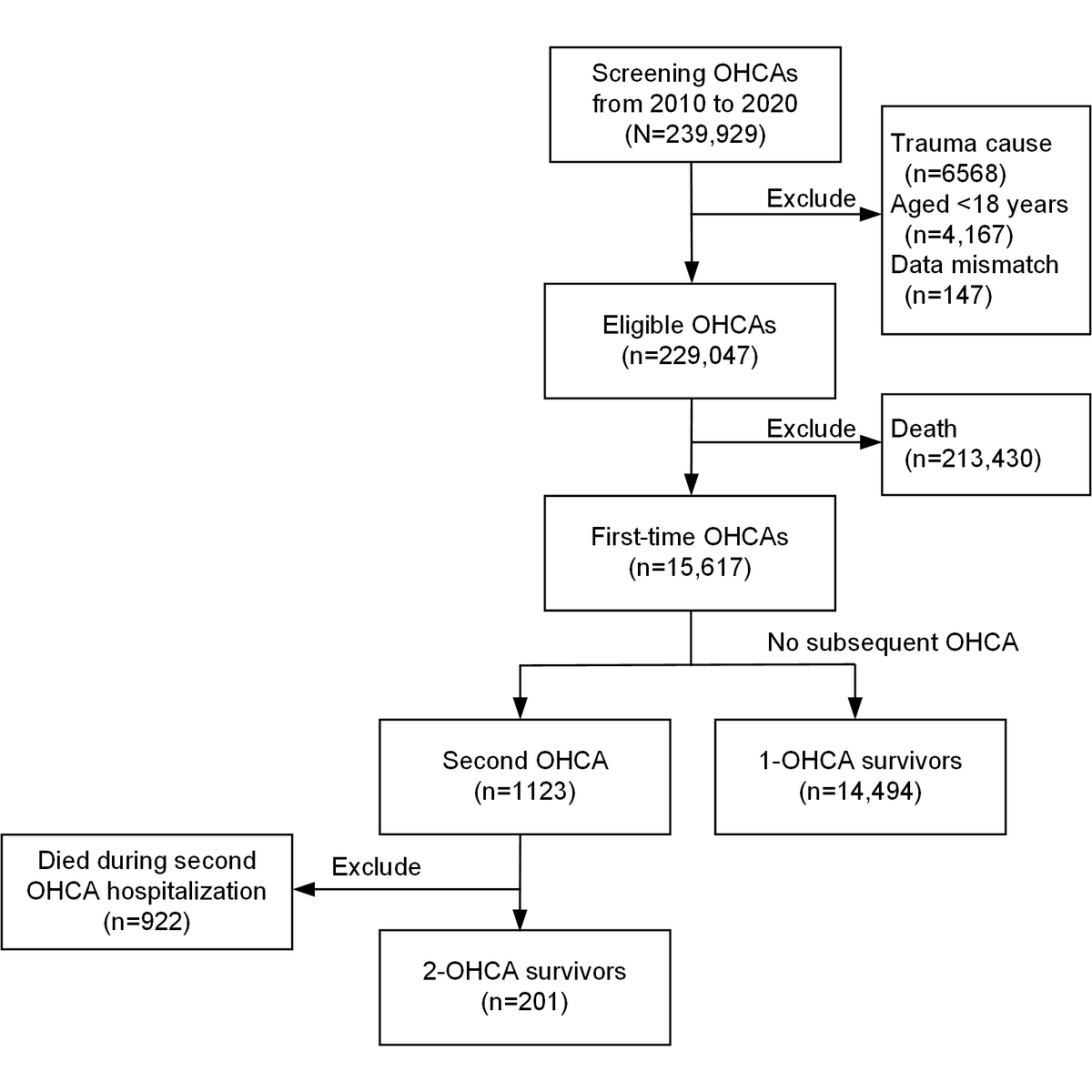
Flow diagram of cohort selection and group classification.

Figure 1 illustrates the patient selection process for the study cohort from 2010 to 2020. Among the 15,617 patients who survived to discharge after a first OHCA, 14,494 had no documented recurrent OHCA during follow-up and were classified as 1-OHCA survivors. A total of 1123 patients experienced a second OHCA. After excluding patients who died during the hospitalization for the second OHCA event (n=922), 201 patients who survived to discharge after both OHCA events were classified as 2-OHCA survivors.

### Baseline Characteristics of the Groups

Table 1 presents the demographics, health care use, life-sustaining interventions, and comorbidities between 1-OHCA and 2-OHCA survivors. Patients in the 2-OHCA group were older (mean 63.13, SD 13.64 years vs mean 60.95, SD 17.42 years; *P*=.02), but sex distribution was similar between groups (127/201, 63.18% vs 8563/14,494, 59.08%; *P*=.24). Use of health care facilities was consistently higher in the 2-OHCA group, including ICU admission (103/201, 51.24% vs 4897/14,494, 33.79%; *P*<.001) and ED visits and/or ward hospitalizations >2 times (117/201, 58.21% vs 7351/14,494, 50.72%; *P*=.03). The rate of life-sustaining device use was significantly higher in the 2-OHCA cohort: nasogastric tube (148/201, 73.63% vs 6752/14,494, 46.58%; *P*<.001), Foley catheter (138/201, 68.66% vs 6339/14,494, 43.74%; *P*<.001), and tracheostomy with ventilator support (92/201, 45.77% vs 3185/14,494, 21.97%; *P*<.001). However, no significant differences between the groups were found in preexisting comorbidities such as diabetes mellitus, hypertension, chronic kidney disease, and liver cirrhosis.

**Table 1:** Comparison of characteristics between 1 out-of-hospital cardiac arrest (OHCA) and 2-OHCA survivors^a^.

Variables		1-OHCA survivors (n=14,494)	2-OHCA survivors (n=201)	*P* value
**Demographics**				
	Age (y), mean (SD)	60.95 (17.42)	63.13 (13.64)	.02
	Sex, male, n (%)	8563 (59.08)	127 (63.18)	.24
**Health care use, n (%)**				
	ICU^b^ admission^c^	4897 (33.79)	103 (51.24)	<.001
	Hemodialysis	752 (5.19)	13 (6.47)	.41
	Outpatient clinics >3^d^	8707 (60.07)	98 (48.76)	.001
	ED^e^ visits and/or ward hospitalizations >2^d^	7351 (50.72)	117 (58.21)	.03
**Life-sustaining indwelling catheter, n (%)**				
	Nasogastric tube	6752 (46.58)	148 (73.63)	<.001
	Foley catheter	6339 (43.74)	138 (68.66)	<.001
	Tracheostomy and ventilator support	3185 (21.97)	92 (45.77)	<.001
**Preexisting comorbidities, n (%)**				
	Diabetes mellitus	2931 (20.22)	46 (22.89)	.35
	Hypertension	4167 (28.75)	68 (33.83)	.11
	Hepatitis	1780 (12.28)	27 (13.43)	.13
	Chronic kidney disease	3485 (24.04)	50 (24.88)	.17
	Asthma	427 (2.95)	6 (2.99)	.97
	Peptic ulcer disease	1027 (7.09)	15 (7.46)	.83
	Heart disease	2737 (18.88)	42 (20.9)	.47
	Cancers	506 (3.49)	5 (2.49)	.44
	Cerebrovascular disease	1243 (8.58)	14 (6.97)	.41
	Hyperlipidemia	1724 (11.89)	25 (12.44)	.81
	Liver cirrhosis	539 (3.72)	8 (3.98)	.50

^a^The qualifying discharge date was discharge after the first OHCA for 1-OHCA survivors and discharge after the second OHCA for 2-OHCA survivors. 1-OHCA survivors: survivors with only one OHCA event. 2-OHCA survivors: survivors with exact 2 OHCA events.

^b^ICU: intensive care unit.

^c^Assessed within the 365 days before the qualifying discharge date.

^d^Assessed within the 30 days before the qualifying discharge date.

^e^ED: emergency department.

### Multivariable Cox Regression Models for Postdischarge Mortality

Table 2 summarizes the multivariable Cox regression analyses evaluating factors associated with postdischarge mortality separately among 1-OHCA and 2-OHCA survivors. In the 1-OHCA group, mortality risk increased with older age (aHR 1.033 per year, 95% CI 1.031-1.035; *P*<.001), male sex (aHR 1.332, 1.268-1.399; *P*<.001), nasogastric tube placement (aHR 2.839, 95% CI 2.622-3.074; *P*<.001), diabetes mellitus (aHR 1.248, 95% CI 1.172-1.328; *P*<.001), chronic kidney disease (aHR 1.358, 95% CI 1.254-1.471; *P*<.001), and cancer (aHR 1.268, 95% CI 1.136-1.414; *P*<.001). Conversely, >3 outpatient visits within 30 days (aHR 0.236, 95% CI 0.224-0.249; *P*<.001), Foley catheter use (aHR 0.905, 95% CI 0.842-0.972; *P*=.006), and tracheostomy or ventilator support (aHR 0.786, 95% CI 0.742-0.832; *P*<.001) were associated with lower observed postdischarge mortality in the multivariable model. These associations should be interpreted as statistical associations rather than evidence of causal protection.

**Table 2:** Factors associated with postdischarge mortality among 1 out-of-hospital cardiac arrest (OHCA) and 2-OHCA survivors^a^.

Variables			1-OHCA survivors				2-OHCA survivors		
			aHR^b^ (95% CI)		*P* value		aHR (95% CI)		*P* value
**Demographics**									
	Age (y)	1.033 (1.031-1.035)		<.001		Not included^c^		Not included	
	Sex, male	1.332 (1.268-1.399)		<.001		Not included		Not included	
**Health care use**									
	ICU^d^ admission^e^	Not included		Not included		Not included		Not included	
	Hemodialysis	1.061 (0.941-1.196)		.33		Not included		Not included	
	Outpatient clinics >3^f^	0.236 (0.224-0.249)		<.001		0.356 (0.259-0.488)		<.001	
	ED^g^ visits and/or ward hospitalizations >2^f^	Not included		Not included		Not included		Not included	
**Life-sustaining indwelling catheter**									
	Nasogastric tube	2.839 (2.622-3.074)		<.001		1.210 (0.699-2.097)		.49	
	Foley catheter	0.905 (0.842-0.972)		.006		0.469 (0.277-0.795)		.005	
	Tracheostomy and ventilator support	0.786 (0.742-0.832)		<.001		0.563 (0.392-0.809)		.002	
**Pre-existing comorbidities**									
	Diabetes mellitus	1.248 (1.172-1.328)		<.001		Not included		Not included	
	Hypertension	0.977 (0.923-1.034)		.42		Not included		Not included	
	Hepatitis	Not included		Not included		Not included		Not included	
	Chronic kidney disease	1.358 (1.254-1.471)		<.001		1.146 (0.725-1.813)		.55	
	Asthma	1.002 (0.881-1.141)		.97		Not included		Not included	
	Peptic ulcer disease	1.038 (0.954-1.131)		.38		Not included		Not included	
	Heart disease	0.998 (0.939-1.062)		.96		Not included		Not included	
	Cancers	1.268 (1.136-1.414)		<.001		Not included		Not included	
	Cerebrovascular disease	1.043 (0.968-1.123)		.26		Not included		Not included	
	Hyperlipidemia	0.938 (0.869-1.012)		.10		Not included		Not included	
	Liver cirrhosis	Not included		Not included		Not included		Not included	

^a^The qualifying discharge date was discharge after the first OHCA for 1-OHCA survivors and discharge after the second OHCA for 2-OHCA survivors. 1-OHCA survivors: survivors with only 1 OHCA event. 2-OHCA survivors: survivors with exact 2 OHCA events. Variables were retained in each multivariable Cox model based on univariate screening, clinical relevance, and model stability. Sparse variables with unstable estimates were not included in the final 2-OHCA model.

^b^aHR: adjusted hazard ratio.

^c^Indicates variables not retained in the final multivariable Cox model.

^d^ICU: intensive care unit.

^e^Assessed within the 365 days before the qualifying discharge date.

^f^Assessed within the 30 days before the qualifying discharge date.

^g^ED: emergency department.

In the 2-OHCA group, although age and sex were clinically important covariates, they were not significantly associated with postdischarge mortality in the univariate Cox regression analysis among 2-OHCA survivors. Given the limited sample size of this subgroup, we did not force these variables into the final model to avoid overfitting and unstable estimates. Lower observed postdischarge mortality was associated with >3 outpatient visits (aHR 0.356, 95% CI 0.259-0.488; *P*<.001), Foley catheter use (aHR 0.469, 95% CI 0.277-0.795; *P*=.005), and tracheostomy or ventilator support (aHR 0.563, 95% CI 0.392-0.809; *P*=.002). No comorbidity was significantly associated with mortality in this subgroup.

The final Cox model for 1-OHCA survivors included 6850 mortality events among 14,494 patients (47.3%). The final Cox model for 2-OHCA survivors included 191 mortality events among 201 patients (95.0%). As the 2-OHCA survivor subgroup had a small overall sample size despite a high number of mortality events, we used a parsimonious modeling strategy to reduce overfitting and model instability. Variables with sparse distributions, complete or near-complete separation, or unstable estimates were not forced into the final 2-OHCA model.

### Survivors of 2-OHCA Had Significantly Worse Long-Term Survival

**Figure 2:**
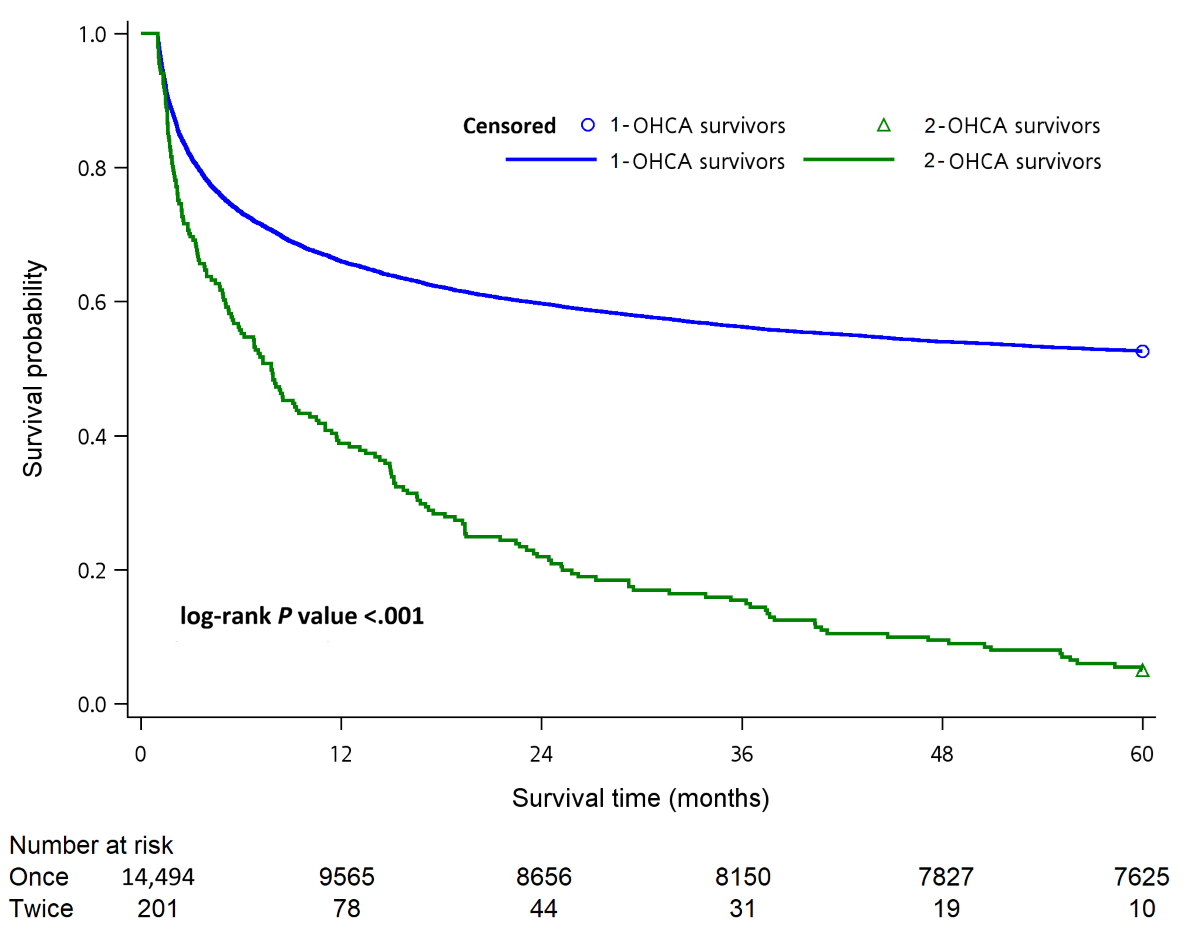
Kaplan-Meier survival curves for 1 out-of-hospital cardiac arrest (OHCA) and 2-OHCA survivors.

Figure 2 shows the Kaplan-Meier curves describing survival after discharge from the qualifying OHCA event. For 1-OHCA survivors, follow-up began at discharge after the first OHCA; for 2-OHCA survivors, follow-up began at discharge after the second OHCA. The 1-OHCA survivor cohort demonstrated a consistently higher postdischarge survival probability than the 2-OHCA survivor cohort. The survival curve of the 2-OHCA group declined steeply during the first year after discharge from the second OHCA. By 60 months, survival remained substantially lower in the 2-OHCA group than in the 1-OHCA group. The log-rank test showed a statistically significant difference between the survivor-defined groups (*P*<.001). As 2-OHCA group membership is conditional on surviving the second OHCA hospitalization, this comparison should be interpreted as a descriptive prognostic comparison rather than a causal estimate of the effect of recurrent OHCA.

### The Distribution of Life-Sustaining Indwelling Device Patterns Among Survivors

Table 3 compares patterns of life-sustaining indwelling device use between 1-OHCA and 2-OHCA survivors. The rate of only Foley catheter use was lower in the 2-OHCA survivors compared with 1-OHCA survivors (5/201, 2.49% vs 704/14,494, 4.86%; *P*=.02). The rate of combined nasogastric tube and Foley catheter use was higher in 2-OHCA survivors (50/201, 24.88% vs 2938/14,494, 20.27%; *P*=.02). Most notably, the rate of using all 3 indwelling devices was significantly higher in 2-OHCA survivors (81/201, 40.30% vs 2642/14,494, 18.23%; *P*<.001), while the rate of patients requiring none of the catheters was markedly lower (45/201, 22.39% vs 6922/14,494, 47.76%; *P*<.001). Other patterns, including nasogastric tube-only, ventilator-only, and 2-device combinations, did not significantly differ between the groups.

**Table 3:** Patterns of life-sustaining indwelling catheter in 1 out-of-hospital cardiac arrest (OHCA) versus 2-OHCA survivors.

	1-OHCA survivors, n (%)	2-OHCA survivors, n (%)	*P* value
Only nasogastric tube	745 (5.14)	9 (4.48)	.55
Only Foley catheter	704 (4.86)	5 (2.49)	.02
Only ventilator	61 (0.42)	1 (0.50)	.81
Nasogastric tube combined with Foley catheter	2938 (20.27)	50 (24.88)	.02
Foley catheter combined with ventilator	55 (0.38)	2 (1.00)	.05
Nasogastric tube combined with ventilator	427 (2.95)	8 (3.98)	.22
Combination of all 3 life-sustaining indwelling catheters	2642 (18.23)	81 (40.30)	<.001
None of the above	6922 (47.76)	45 (22.39)	<.001

## Discussion

### Principal Findings

In this nationwide cohort, patients who survived discharge after a second OHCA had substantially poorer subsequent postdischarge survival than patients who survived a single OHCA and had no documented recurrence. As 2-OHCA survivor status was conditional on survival after the first OHCA, occurrence of a second OHCA, and survival to discharge after the second event, this comparison should be interpreted as a descriptive postdischarge prognostic comparison between survivor-defined groups rather than as evidence that the second OHCA itself caused poorer long-term survival. Moreover, the factors statistically associated with mortality in this group differed from those observed in 1-OHCA survivors and included claims-based indicators of life-sustaining device use. These device-related variables should be interpreted as markers of postdischarge dependency, care setting, and survivorship rather than as causal determinants of survival. Marked disparities in device dependence were also observed, whereas a substantial proportion of 1-OHCA survivors lived without supportive devices, and fewer than one-fourth of 2-OHCA survivors achieved device-free survival.

### Clinical Registry Versus Population-Level Registry

The study by Awad et al [19] examined 1-year survival among hospital discharge OHCA survivors using linked clinical registry and administrative data in British Columbia and were able to adjust for key OHCA characteristics and prehospital variables, including arrest location, witnessed status, bystander cardiopulmonary resuscitation, initial rhythm, and in-hospital interventions. In contrast, the NHIRD provides nationwide longitudinal coverage that allowed us to identify the extremely rare 2-OHCA survivor population, but it lacks granular resuscitation variables and direct neurological outcomes. This contrast highlights the trade-off between clinical granularity and population-level scale. Additionally, these findings highlight the value of population-level claims data for postarrest surveillance, particularly for rare survivor-defined groups that are difficult to evaluate in traditional clinical datasets.

### Explaining the Divergent Regression Models

The 2-OHCA survivors in our cohort constitute an even rarer, doubly selected population: those who survived the initial OHCA experienced a subsequent event and remained observable during the 5-year follow-up. This conditioning introduces a “depletion of susceptibles” effect [20] and attenuates the prognostic value of baseline comorbidities. Accordingly, traditional covariates (eg, age, male sex, diabetes, chronic kidney disease, and cancer) maintain strong associations with mortality in 1-OHCA survivors, whereas in 2-OHCA survivors, these associations are largely diminished or no longer significant. Instead, the current physiological status and context of care have become dominant determinants.

The pattern of device-related variables illustrates this shift. Among 1-OHCA survivors, nasogastric tube use is independently associated with higher mortality, likely reflecting dysphagia, frailty, and aspiration risk. In contrast, among 2-OHCA survivors, the prognostic impact of nasogastric tube use disappears, and survival is more closely associated with stable, organized support systems. Notably, the inverse associations observed for Foley catheter use and tracheostomy with ventilator support should not be interpreted as evidence that device use causally reduces mortality. More plausibly, these device-related variables reflect survivorship conditioning and postdischarge care context. Patients who were stable enough to receive long-term device support and survive to hospital discharge had already passed through the highest-risk phase after OHCA, whereas patients who died early would not have been observed as chronic device users in the postdischarge setting. Therefore, Foley catheter use and tracheostomy with ventilator support may serve as claims-based markers of survivorship rather than protective interventions.

### Increased Device Dependence After a Second OHCA

We also observed a significant shift toward increased dependence on life-sustaining indwelling devices in the 2-OHCA group. Single device use has been increasingly replaced by the concurrent use of multiple devices. Among the few individuals who remained device-free after recurrence, the absence of life-sustaining indwelling devices may indicate lower postdischarge dependency. However, because neurological outcomes were not available in the NHIRD, favorable neurological recovery cannot be directly inferred from these claims-based indicators. These observations are consistent with the regression results: in 2-OHCA survivors, mortality risk was more closely associated with current support status rather than with baseline comorbidities.

The distribution of device use patterns significantly differed between 1-OHCA and 2-OHCA survivors. Device dependence is an informative claims-based marker of ongoing vulnerability among 2-OHCA survivors. However, causality cannot be inferred. The prominence of device-related variables in the regression models may reflect disability severity, survivorship, care setting, care intensity, or unmeasured confounding rather than a direct effect of the devices themselves. These findings suggest that postdischarge dependency and care needs should be incorporated into risk stratification and follow-up planning, but they should not be interpreted as evidence that device placement is beneficial. Our findings highlight the importance of incorporating the device status into post-OHCA risk stratification and follow-up planning. The underlying mechanisms linking device dependence, care setting, and long-term survival require further investigation.

### Limitations

In this nationwide, population-based dataset, the scale and completeness enabled survival analyses that are rarely feasible even in large multicenter cohorts and clarified how risk structures differ between 1-OHCA and 2-OHCA cohorts. However, several methodological limitations should be emphasized. First, classification as a 2-OHCA survivor was conditional on surviving long enough after the first OHCA to experience a second OHCA and subsequently survive to discharge after the second event. Therefore, 2-OHCA status is inherently time-dependent, and comparison with 1-OHCA survivors may be affected by selection bias, survivorship conditioning, and guarantee-time or immortal-time-like bias. Accordingly, the Kaplan-Meier curves were interpreted as descriptive postdischarge survival trajectories after the qualifying OHCA event, rather than as causal estimates of the effect of a second OHCA. Second, device-related variables were derived from administrative claims and should be interpreted as surrogate indicators of functional dependency, care setting, care intensity, or unmeasured clinical status, rather than as direct measures of physiological benefit or care quality. Therefore, the inverse associations observed for Foley catheter use and tracheostomy or ventilator support should not be interpreted as causal or protective effects of device use. Third, the 2-OHCA survivor group was small, which limited statistical precision and increased the risk of sparse data bias, model instability, and overfitting. The absence of statistically significant associations for baseline comorbidities should not be interpreted as evidence that these conditions are clinically irrelevant, and the subgroup-specific Cox model should be viewed as exploratory and hypothesis-generating. Future studies using larger datasets, time-dependent Cox models, landmark analyses, or multistate models are needed to validate these findings and better estimate the dynamic effect of recurrent OHCA. Finally, confounding by indication is also possible, particularly for device-related variables. For example, tracheostomy with ventilator support may represent necessary ongoing life-sustaining care among chronically dependent patients rather than a directly protective intervention. Additionally, we did not have the granular OHCA clinical variables in the NHIRD, including initial rhythm, witnessed status, bystander cardiopulmonary resuscitation, prehospital resuscitation details, no-flow or low-flow time, and neurological outcome at discharge. These variables are among the strongest predictors of survival after cardiac arrest and could confound the observed associations between comorbidities, device-related variables, and mortality. It is a trade-off because the strength of the NHIRD is its nationwide coverage over a 10-year period, which enabled the identification of the extremely rare group of patients who survived exactly 2 OHCA episodes; however, this population-level scale comes at the cost of limited clinical granularity.

### Conclusions

In this nationwide cohort, patients who survived discharge after a second OHCA represented a rare, highly selected, and clinically vulnerable survivor population with poor subsequent 5-year survival. In exploratory subgroup analyses, mortality among 2-OHCA survivors appeared to be more closely associated with life-sustaining device status than with baseline comorbidities; however, this finding should be interpreted cautiously given the small subgroup size, model-selection constraints, and potential sparse data bias.

## Data Availability

The individual-level National Health Insurance Research Database data used in this study are subject to governmental data access restrictions and privacy regulations. These data are not publicly available and cannot be distributed by the authors. Access requires an application to and approval by the appropriate data governance authority in Taiwan. Aggregated results or analytic code may be available from the corresponding author upon reasonable request, where permitted by regulations.

## Abbreviations

aHR: adjusted hazard ratio
ED: emergency department
ICD: International Classification of Diseases
ICU: intensive care unit
NHIRD: National Health Insurance Research Database
OHCA: out-of-hospital cardiac arrest
